# Furthering Our Understanding of Post-Traumatic Mental Health Conditions and Intimate Relationship Outcomes in Veterans of the Wars in Afghanistan and Iraq

**DOI:** 10.3390/bs15060719

**Published:** 2025-05-23

**Authors:** Camara A. T. Azubuike, Alexander O. Crenshaw, Candice M. Monson

**Affiliations:** 1 Department of Applied Psychology and Human Development, University of Toronto, Toronto, ON M5S 1A1, Canada; c.azubuike@mail.utoronto.ca; 2 Department of Psychological Science, Kennesaw State University, Kennesaw, GA 30144, USA; acrens13@kennesaw.edu; 3 National Center for PTSD Women’s Health Sciences Division, VA Boston Healthcare System, Boston, MA 02130, USA; 4 Department of Psychiatry, Chobanian & Avedisian School of Medicine, Boston University, Boston, MA 02118, USA

**Keywords:** PTSD, veterans, depression, alcohol, relationship adjustment

## Abstract

Objective: Although there has been substantial research on post-traumatic stress disorder and its commonly comorbid conditions, the unique associations among these mental health symptoms and relationship adjustment have not been investigated. The purpose of this paper is to extend understanding of the associations among PTSD and relationship adjustment for female and male veterans, as well as to account for the impact of comorbid symptoms of depression and problematic alcohol use in a large sample. Method: Participants were 2325 (*n* = 1122 men and 1203 women) veterans of the wars in Iraq and Afghanistan from a larger study exploring wartime experiences and post-deployment adjustment. Chi-square analyses and regressions tested the associations among mental health symptoms (PTSD symptom severity, depressive symptoms, and problematic alcohol use) and relationship status and adjustment. Results: For both men and women, those with probable PTSD were less likely to be in an intimate relationship than those without probable PTSD, and those in intimate relationships had lower PTSD symptom severity compared with those not in intimate relationships. However, when accounting for PTSD, depression, and problematic alcohol use simultaneously, only depression emerged as a significant negative predictor of relationship adjustment. Conclusions: Shared variance among these common post-traumatic mental health conditions comorbidities may be most responsible for PTSD’s negative association with relationship adjustment. Unique remaining variance of depression is also negatively associated with relationship adjustment. Treatment implications of these findings for individual and couple therapy post-trauma are provided.

## 1. Furthering Our Understanding of Post-Traumatic Mental Health Conditions and Intimate Relationship Outcomes in Veterans of the Wars in Afghanistan and Iraq

Post-traumatic stress disorder (PTSD) is linked with poor intimate relationship adjustment for those in a relationship (McGinn et al., 2017). However, conditions commonly comorbid with PTSD (e.g., depression and substance use) also occur after trauma exposure (Debell et al., 2014) and have well-established associations with relationship problems (Goldfarb & Trudel, 2019). It is unknown if associations between PTSD and relationship adjustment are uniquely attributable to PTSD, better attributable to other conditions, or attributable to shared variance among PTSD and other conditions. This study seeks to answer some remaining questions in the field of traumatic stress and intimate relationship functioning in a large sample of male and female veterans from Iraq and Afghanistan. These questions include the likelihood of being in an intimate relationship based on probable PTSD status and PTSD symptom severity based on relationship status. Moreover, the current study seeks to identify common post-traumatic mental health symptoms most relevant to relationship adjustment.

Research has consistently documented negative associations between PTSD symptoms and relationship adjustment, especially among military samples (e.g., Monson et al., 2009; Taft et al., 2011). Using the sample of interest to the current study, Zelkowitz et al. (2023) found a negative association between PTSD symptoms and relationship adjustment, with no differences in the strength of the association for men and women. Meanwhile, epidemiological and observational studies demonstrate that up to half of those with PTSD also suffer from depression (Pietrzak et al., 2011; Rytwinski et al., 2013). Depression is also consistently associated with intimate relationship adjustment cross-sectionally and longitudinally (e.g., Gustavson et al., 2012; Kouros et al., 2008; Najman et al., 2014; Whisman et al., 2021). Similarly, research consistently demonstrates high rates of co-occurring substance use disorders and PTSD (e.g., Debell et al., 2014; Fox et al., 2020), and between substance use problems and relationship adjustment (Fischer & Wiersma, 2012; Holden & Rollins, 2019).

These well-established findings raise questions about the association between PTSD symptoms and having an intimate relationship, and exactly which trauma sequelae are responsible for relationship problems and vice versa. Based on the available epidemiological data (Pietrzak et al., 2011), we hypothesized that both men and women with a probable diagnosis of PTSD would be less likely to be in an intimate relationship. Given the well-documented negative association between PTSD and relationship adjustment, we hypothesized that this association would remain significant when accounting for depression symptoms and problematic alcohol use problems. We also examined potential gender differences in these associations.

## 2. Method

### 2.1. Participants

Participants were 2325 (*n* = 1122 men and 1203 women) veterans deployed in support of the wars in Iraq and Afghanistan, who participated in a larger study investigating wartime experiences and post-deployment adjustment (Street et al., 2013). Potential participants were randomly selected from the Department of Defense Manpower Data Center’s roster of Operation Enduring Freedom/Operation Iraqi Freedom Veterans separated from active-duty service. Random sampling was conducted separately for men and women, with female veterans oversampled to allow for similar numbers of men and women. From an initial sampling frame of 6000 veterans, 940 did not have a valid address, 123 were ineligible (i.e., deceased, currently deployed, never deployed in support of wars in Iraq/Afghanistan), and 213 declined participation. From 2348 participants who completed the survey (response rate = 48.6%), 2325 responded to questions regarding intimate relationships. Overall, 77% of participants (*n* = 1791) self-reported their relationship status on a demographic form as being in an intimate relationship (men = 80%, women = 75%, gender difference *X*^2^ (1, *n* = 2325) = 8.59, *p* < 0.01). Participant demographic and military service characteristics, stratified by gender are presented in Table 1.

### 2.2. Procedure

Data collection occurred between September 2009 and November 2010 through a multi-stage mailing procedure, including a personalized introductory letter followed one week later by another letter, the survey, and a 5 USD cash incentive. Non-responders received a postcard reminder, followed by a second survey two weeks later, and a third survey three weeks after that. A fact sheet detailing the elements of informed consent was included with each survey. All materials were approved by the Boston VA Medical Center Institutional Review Board.

### 2.3. Measures

The Dyadic Adjustment Scale-Seven items (DAS-7, Hunsley et al., 2001) measured relationship adjustment, which is the overall quality and functioning of a relationship. Scores below 21.5 indicate clinically significant relationship distress (Funk & Rogge, 2007). For the present study, Cronbach’s α = 0.87. Only participants who indicated they were currently in an intimate relationship completed this measure.

The post-traumatic stress disorder checklist (PCL; Weathers et al., 1993) is a self-report questionnaire that measures the severity of each Diagnostic and Statistical Manual of Mental Disorders, Fourth Edition, Text Revision (DSM-IV-TR) PTSD symptom over the past month. This 17-item measure uses a Likert scale response from 1 (not at all) to 5 (extremely). The PCL has demonstrated excellent internal consistency, along with sensitivity and specificity for PTSD diagnosis (e.g., Weathers et al., 1993). We used the recommended and conservative cut-off score of 45 to develop a dichotomous probable PTSD/no PTSD variable (Weathers et al., 1993; Hotopf et al., 2006). For the present study, Cronbach’s α = 0.97.

Depression symptoms were measured using the 10-item Boston version of the Center for Epidemiologic Studies Depression Scale (CES-D; Radloff, 1977), which assesses depressive symptoms in the past week using a Likert response scale ranging from 1 (“None of the time or less than one day”) to 4 (“5–7 days”). A cutoff of 10 was used to categorize participants into probable depression/no depression groups (Andresen et al., 1994). For the present study, Cronbach’s α = 0.90.

The CAGE questionnaire (Ewing, 1984) assessed problematic alcohol use. Participants were instructed to select “Yes” or “No” to four questions about their drinking behavior (e.g., “Have you felt you ought to cut down on your drinking?”). As with prior studies, a score of 2 was used to categorize participants into problematic alcohol use/no problematic alcohol use groups (Buchsbaum et al., 1991). For the present study, Cronbach’s α = 0.74.

### 2.4. Statistical Analyses

We conducted chi-square analyses to examine the association between dichotomous relationship status (i.e., in an intimate relationship vs. not in a relationship) and probable PTSD diagnosis, separately for men and women. To test whether this association differed by gender, we conducted a logistic regression with gender, probable PTSD diagnosis, and their interaction predicting relationship status. Among participants who reported being in an intimate relationship, we used linear regression to examine the association between PTSD symptom severity and relationship status, testing for potential gender differences in this relationship by including a gender × relationship status interaction term. Finally, to examine the association between PTSD symptoms and relationship adjustment, while accounting for depression and problematic alcohol use, we conducted multiple regression analyses for men and women separately from this large sample. We then conducted a linear regression, including interactions of each mental health symptom by gender to test for potential gender effects.

## 3. Results

In examining the association between probable PTSD and intimate relationship status, those with probable PTSD were less likely to be in an intimate relationship than those without probable PTSD in both men, χ^2^ [1, *n* = 1106] = 6.29, *p* = 0.012, and women, χ^2^ [1, *n* = 1172] = 6.80, *p* = 0.009. For men, 70% with probable PTSD were in an intimate relationship, and 77% without probable PTSD were in an intimate relationship. For women, 60% with probable PTSD were in an intimate relationship, and 68% without probable PTSD were in an intimate relationship. The association between probable PTSD and intimate relationship status did not significantly differ in gender in the logistic regression (OR = 1.02 [0.675, 1.533], *p* = 0.936). Moreover, linear regression revealed that being in an intimate relationship was associated with lower PTSD symptom severity (*B* = −3.05, *p* < 0.01); this association did not significantly differ in gender (*B* = 0.73, *p* = 0.665).

When examining associations among PTSD symptom severity and relationship adjustment, while accounting for depression and problematic alcohol use, depression emerged as the only significant predictor of relationship adjustment for both men and women (see Table 2). Neither PTSD nor problematic alcohol use symptoms were significantly associated with relationship adjustment for men or women in these analyses. In the follow-up linear regression testing all gender by mental health symptom interactions in predicting relationship adjustment, there were no differences in gender (*p*s = 0.098–0.794).

## 4. Discussion

The current study extended our understanding of the association between PTSD and relationship outcomes by examining the association between PTSD and intimate relationship status, and how the well-documented association between PTSD and relationship problems is potentially affected by common comorbid mental health symptoms. We used a large, gender-balanced sample of US veterans of the wars in Iraq and Afghanistan, which allowed us to more confidently assess these interrelationships. Consistent with hypothesis and the scant epidemiological data on the topic (e.g., Whisman, 2001), men and women with a probable PTSD were less likely to be in an intimate relationship. Moreover, those in an intimate relationship had lower levels of PTSD symptoms. There were no gender differences in any of these associations.

Contrary to hypothesis, when depression and problematic alcohol use symptoms were simultaneously included in the model with PTSD symptoms predicting relationship adjustment, only depression emerged as a significant negative predictor of relationship adjustment. This pattern of results suggests that each of these common trauma sequelae may not show the unique and independent associations with relationship adjustment; rather, they suggest associations are either primarily attributable to depression or to shared variance between PTSD, depression, and problematic alcohol use. Previous research has indeed found that PTSD and depression have a considerable overlap in symptoms and that the shared variance between PTSD and depression is mainly attributable to PTSD symptoms involved in arousal/reactivity and negative alterations in cognitions and mood (Contractor et al., 2018). Likewise, problematic alcohol use may not have had an independent association with relationship adjustment because of the overlapping association with the hyperarousal and avoidance symptoms, consistent with the self-medication hypothesis of the association between PTSD and problematic substance use (María-Ríos & Morrow, 2020). Our results may therefore be most consistent with the “shared variance” explanation of comorbidity and relationship adjustment.

It is important to note that this study relies on self-report data to assess symptoms of PTSD, depression, and problematic alcohol use, raising possible concerns about shared method variance (Spector et al., 2019). Additionally, the current study used a measure of PTSD consistent with the DSM-IV-TR (American Psychiatric Association, 2000), which has somewhat different criteria to DSM-V-TR (American Psychiatric Association, 2022), and therefore results with the current taxonomy may vary. Additionally, this study did not assess a range of medical conditions found in this population that may account for the PTSD symptom presentation, such as mefloquine or traumatic brain injury exposure (Bryant, 2011; Nevin & Ritchie, 2015) or other mental health conditions such as bipolar, generalized anxiety, or panic disorders (e.g., Brady et al., 2000).

These findings point to an additional potential pathway for improving relationship functioning in those who have been traumatized. A shared decision-making approach that includes options for individual evidence-based psychosocial and/or pharmacological treatment to improve depressive symptoms overlapping with PTSD and alcohol misuse may lead to improvements in relationship functioning. Moreover, while there are PTSD-focused couple therapies for PTSD and relationship enhancement (e.g., Monson & Fredman, 2012), depression-focused couple therapy (e.g., Baucom et al., 2018) might be a viable and efficient approach to improving individual mental health problems and relationship functioning. Existing individual and conjoint PTSD treatments might also be enhanced by specific attention to depressive symptoms. Future research should explore these associations using broader substance use measures (e.g., cannabis and opioids) and behavioral addictions (e.g., gambling) to determine if similar or distinct patterns emerge.

## 5. Conclusions

The current study extends our understanding of the associations between PTSD, depression, problematic alcohol use, and relationship adjustment in a large sample of veterans from Iraq and Afghanistan. Consistent with expectations, individuals with probable PTSD were less likely to be in an intimate relationship, and those in intimate relationships reported lower PTSD symptom severity. However, when accounting for comorbid symptoms, only depression emerged as a significant predictor of relationship adjustment. These findings suggest that shared variance among PTSD, depression, and problematic alcohol use may primarily account for PTSD’s association with relationship functioning. Given the substantial overlap between PTSD and depression, particularly in arousal/reactivity and negative alterations in cognition and mood, interventions targeting depressive symptoms may be particularly beneficial for improving both individual mental health and relationship adjustment. Findings have implications for both individual and couple-based interventions post-trauma, highlighting the need for treatments that explicitly address depressive symptoms to enhance relationship outcomes.

## Figures and Tables

**Table 1:** Full sample of demographic and military service characteristics divided by gender.

	Females (*n* = 1209)	Males (*n* = 1139)
Age M (SD) ^b^	34 (8.9)	37 (10)
Race ^b^		
White	70.9%	83.8%
African American	20.8%	8.8%
Other	8.3%	7.4%
Number of Deployments to the Wars in Afghanistan and Iraq		
M (SD) ^b^	1.3 (0.65)	1.5 (0.76)
Total Number of Months Deployed to the Wars in Afghanistan and Iraq		
M (SD) ^b^	11 (7.4)	12 (8.4)
Location of Deployment:		
% Deployed to Iraq ^b^	57.4%	68.7%
% Deployed to Afghanistan	12.2%	14.7%
% Deployed to other locations ^b^	37.2%	28.4%
Military Occupation Specialty:		
% served Combat-Arms ^b^	6.0%	33.2%
% served Combat-Support ^b^	50.8%	44.7%
% served Service-Support ^b^	40.9%	20.8%
Branch of Military Service ^b^		
Marines	4.3%	14.3%
Army	55.3%	51.9%
Navy	15.7%	12.3%
Air Force	24.1%	20.9%
Coast Guard	0.3%	0.1%
Multiple Branches	0.3%	0.5%
Military Service Component		
Reserves/Guard	48.1%	50.8%
Active Duty	50.5%	47.4%
Both Reserves/Guard and Active Duty	1.4%	1.9%

Note. ^b^ Denotes a significant difference between genders, *p* < 0.01.

**Table 2:** Associations among PTSD, depression, and problematic alcohol use symptoms predicting relationship adjustment for men and women veterans in intimate relationships.

	Men			Women		
	B	SE	*p*	B	SE	*p*
Intercept	32.42	0.48	<0.001	32.69	0.48	<0.001
PTSD	0.04	0.02	0.073	0.03	0.02	0.153
Depression	−0.42	0.05	<0.001	−0.39	0.05	<0.001
Problematic Alcohol Use	−0.33	0.20	0.097	0.19	0.24	0.423

Notes. PTSD symptoms were measured with the PTSD checklist (Weathers et al., 1993). Depression symptoms were measured with the center for epidemiologic studies depression scale (CES-D; Radloff, 1977). Problematic alcohol use was measured with the CAGE questionnaire (Ewing, 1984). Relationship adjustment was measured with the dyadic adjustment scale-seven items (Hunsley et al., 2001).

## Data Availability

The datasets used in this article are not readily available because clients did not consent at the time to make their data publicly available. Requests to access the datasets should be directed to Dr. Candice Monson.
